# Attitudes toward and knowledge of collaboration of dental and medical practice among medical students in Southern India: a cross-sectional questionnaire survey

**DOI:** 10.12688/f1000research.111130.4

**Published:** 2023-06-27

**Authors:** Harshit Atul Kumar, Ashita Uppoor, David Kadakampally, B Unnikrishnan, Prasanna Mithra

**Affiliations:** 1 Senior Lecturer, Department of Orthodontics and Dentofacial Orthopedics, Manipal College of Dental Sciences, Mangalore,Manipal Academy of Higher Education, Manipal, Karnataka, 576104, India; 2 Professor and Dean, Department of Periodontology, Manipal College of Dental Sciences, Mangalore, Manipal Academy of Higher Education, Manipal, Karnataka, 576104, India; 3 Associate Professor, Department of Periodontology, Manipal College of Dental Sciences, Mangalore, Manipal Academy of Higher Education, Manipal, Karnataka, 576104, India; 4 Dean and Professor, Department of Community Medicine,, Kasturba Medical College, Mangalore, Manipal Academy of Higher Education, Manipal, Karnataka, 576104, India; 5 Additional Professor, Department of Community Medicine,, Kasturba Medical College, Mangalore Manipal Academy of Higher Education, Manipal, Karnataka, 576104, India

**Keywords:** Dental-Medical Collaboration, Attitude and knowledge, Medical students, Interprofessional practice

## Abstract

Background:

Enhancing oral health care services provided through inter-professional collaboration between medical and dental practitioner is important, and even essential. The purpose of this study is to assess the attitude toward and knowledge of medical-dental collaborative practice among medical students attending colleges in Southern India.

Methods:

A cross sectional questionnaire survey was conducted among medical students and interns of medical colleges in coastal South India with prior information and permission. The questionnaire consisted of 11 questions to assess attitude toward and knowledge of medical-dental collaborative practice. The demographic backgrounds of participants were also recorded. Chi square test was employed for data analysis. The responses obtained were correlated with age, gender and year of study of participants using Pearson’s correlation test

Results:

A total 250 questionnaires were distributed and 234 responses were appropriately completed. Most of the students agreed that oral health was an integral part of systemic health, however participants disagreed on attending compulsory rotation in dentistry at a statistically significant level (p<0.05), moreover participants did not agree with physicians having an active role in motivating their patients for regular dental check-up. 82% of the medical students believe that dental check-up should be included in health packages under health insurance. A statistically significant (p<0.05) difference was observed among 3 ^rd^ year & 4 ^th^year students and interns and also it was found that female students provided more positive responses towards medical-dental collaboration.

Conclusions:

Even though medical students showed fairly positive attitudes and knowledge towards dentistry, the analysis within the study groups showed that knowledge and attitudes regarding the collaborative practice worsened over the academic years among the medical students. In order to destigmatize and foster interdisciplinary collaboration which would contribute to higher resource efficiency and the standard of care, continuing education in both the disciplines would be highly beneficial.

## Introduction

One of the commonly neglected health issues globally is oral health, and the impact of oral diseases has significant effects on individuals, communities and global health care systems. ^1^ The evolution of professional health care education over the years is yet to compete with the demographical challenges and inequalities faced, which causes a burden on the system to fight the disease spread, and utilize the scientific knowledge and advanced technology with increasing complexity in the system. In addition to this, there is existing gap in imparting training to medical professionals about importance of oral health and its impact on general health. ^2^^,^^3^

In order to achieve greater resource efficiency and upgrade the standard of care and comprehensiveness by reducing duplication and gaps in services, interprofessional collaboration is a key to success. ^4^ All parties will benefit from improved professional cooperation between medical and dental practitioners and better educate the public. Overlooking underlying health problems while treating a patient is what most dentists do while they focus on the diagnosis and treatment of oral diseases. Likewise, doctors may fail to notice their patient’s oral health problems which could result in initiation of a long-lasting medical illness. Enhancing health care services through inter-professional collaboration between medical and dental practitioners is therefore essential. ^5^ An article was published after the First Systemic Health Round Table Discussion to advocate for better medical-dental collaborative practice. Inter-professional collaboration enhances communication and decision-making, enabling a synergistic influence of grouped knowledge and skills. ^6^ Due to limited literature and emphasis on this topic, we decided to conduct this study to improve the understanding and importance of the same.

As per the medical education curriculum in India, medical students have to attend compulsory rotation-ship of about one month in a dental clinic/hospital, which by far many medical colleges follow it routinely. Hence the purpose of this study is to evaluate the knowledge and attitudes of the medical students studying in medical college attached to a dental college, towards collaboration between medical and dental practice in South India to understand the shortcomings and address them with a better strategy.

## Methods

### Ethical approval

Approval was obtained from the Institutional ethics committee (IEC) with protocol reference number-17020, from Manipal College of Dental Sciences, Mangalore. Necessary permissions and the written consent of participants were obtained and all methods were in accordance with relevant guidelines and regulations for carrying out the survey.

### Study and questionnaire design

The questions in the questionnaire survey were adapted from the previously published studies and modified as per requirement for this study. ^6^^,^^7^ The questionnaire was pretested in pen and paper format(physical format) on a group of 20 medical students selected based on our inclusion and exclusion criteria and the participants were given brief explanation about the study and the feedback was collected. Based on the feedback from participants, minor corrections were incorporated in the questionnaire and updated questionnaire was used in the main study. This cross-sectional study was carried out among the 3 ^rd^ year, 4 ^th^ year students and interns (5 ^th^ year) of four medical colleges with an attached dental college in and around Mangalore, a coastal urban area in the south Indian state of Karnataka. A total of 900 medical students were invited to participate in the study from all the medical colleges, out of which 250 students consented to participation to fill the questionnaire in the pen and paper format (physical format). From 250 responses, 234 questionnaires were appropriately completed and 16 responses were excluded as they submitted incompletely filled questionnaires. Dental students were not considered to be included in the study because the dental curriculum is designed such that dental students has a good exposure to medical college departments throughout the entire course with mandatory clinical postings and University examination when compared to medical students who only have a month of posting in dental college as required by medical curriculum.

Inclusion criteria:

1. Undergraduate Medical Student studying in 3 ^rd^, 4 ^th^ or 5 ^th^ year of the college.
2. Students who consent to participate in the survey

Exclusion criteria:

1. Medical student studying in 1 ^st^ or 2 ^nd^ year of the college
2. Students belonging to paramedical course in the same college
3. Post graduate medical students

The questionnaire was subjected to face and content validation by both a medical and dental faculty member for its comprehensiveness and simplicity of understanding and each question was tested for content validity.

After obtaining the written consent from the participants, questionnaire to be filled were distributed among the participants. The questionnaire had 2 components: the first component was to collect the demographic data such as age (below 20 years of age, 21-24 years, above 20 years of age), gender (male and female) and year of study (3 ^rd^ year, 4 ^th^ year, interns) and the second component contained 11 objective questions which were designed to assess their attitude and knowledge. Questionnaire responses were recoded using 5-point Likert scale (1-strongly disagree, 2-disagree, 3-neutral, 4-agree, 5-strongly agree).

### Score calculation and interpretation

For the study to be statistically valid and comprehensible, we calculated the mean response score for each question Shapiro wilk test will be used to the test the normality. If the data is skewed, median and inter quartile range will be used for analysis.

The collected data were coded and analysed using statistical package of social sciences (SPSS) version 11.5. Results were expressed as proportion and summary measures (median with inter quartile range) using appropriate tables and figures. For comparison across the groups Mann Whitney U test was employed. A p value of 0.05 was considered statistically significant.

## Results

A total of 900 students were invited to participate in the study, out of which 250 students consented to participate, which amounts to 27.7% of response rate. Although the response rate was less than expected, the statistically calculated required sample size of minimum180 responses was completed.

Reliability analysis was done using Cronbach's alpha and it was found to be 0.769 with no improvement in alpha on deletion of any item in the questionnaire.

Those who returned a blank or incomplete questionnaire were excluded. The mean age of the participants was 21.5 years with 58.36% respondents being female and 41.64% male. Out of total respondents, 43.77% were 3 ^rd^ years, 39.9% were 4 ^th^ years and rest (17%) accounted for interns. Shipiro wilk test for mormality was performed and the data was found to be skewed (p value<0.001). Thus , statistical analysis was performed using non parametric tests.

There was no statistically significant difference in knowledge and attitude based on gender, except that the female students were significantly more (p value-0.00) aware of interprofessional referral practice before elective medical surgeries ( Table 1). Overall analysis of gender-based difference in responses indicated that females are more well informed and have increased positive attitude than males regarding the intended collaboration.

**Table 1.** Responses based on gender.

Characteristic	Gender		Total Median (IQR)	p value
	Male (n=97) Median (IQR)	Female (n=137) Median (IQR)		
Oral health is an integral part of general health	5.0(4.0-5.0)	5.0(4.0-5.0)	5.0(4.0-5.0)	0.989
Periodontitis is the 6 ^th^ complication of diabetes	3.0(3.0-4.0)	4.0(3.0-4.0)	3.0(3.0-4.0)	0.402
Oral check-up for all woman in pre-natal care	4.0(3.0-4.0)	4.0(3.0-4.0)	4.0(3.0-4.0)	0.093
HIV pt. with CD4 count > 200 cells/mm blood is suitable for dental treatment	3.0(3.0-4.0)	3.0(3.0-4.0)	3.0(3.0-4.0)	0.627
Salivary biomarkers used in diagnosis of oral and systemic diseases	4.0(3.5-4.0)	4.0	4.0	0.642
Physician should advice and motivate their patients to undergo dental check-up regularly	4.0(4.0-5.0)	5.0(4.0-5.0)	4.0(4.0-5.0)	0.117
Medical students should attend compulsory rotation in dentistry	4.0(3.0-4.0)	4.0(3.0-4.0)	4.0(3.0-4.0)	0.716
Complete dental check-up should be covered in health insurance	4.0(3.0-5.0)	4.0(4.0-5.0)	4.0(4.0-5.0)	0.238
Referral for dental check-up before any elective surgeries	3.0(3.0-4.0)	4.0(3.0-4.0)	4.0(3.0-4.0)	0.000 *
Integral collaboration between medicine and dentistry	4.0(3.0-4.0)	4.0(3.0-4.0)	4.0(3.0-4.0)	0.809
Interact with dental students and mutually exchange knowledge	4.0(3.0-4.0)	4.0(3.0-4.0)	4.0(3.0-4.0)	0.997

* p=0.05.

Most of the students agreed that oral health was an integral part of systemic health with analysis leading to median score of 5 ( Table 2), but a statistically significant difference in the attitudes of medical students based on study year was seen when asked about attending compulsory rotation in dentistry with senior students showing negative attitude. The majority of participants had adequate knowledge regarding the medical-dental relationship, but almost 47% had very limited awareness about the existing relationship, which was assessed by questions 2, 3, 4 and 5. While comparing the groups based on study year there was a statistically significant difference in knowledge ( Table 2) with p value of 0.003, when asked about importance of salivary bio markers. Moreover when asked about physicians’ active role in motivating their patients for regular dental check-up, there was a statistically significant difference (p value-0.013) in responses given by groups based on year of study indicating their negative attitude. However, 82% of the medical students were of the opinion that dental check-ups should be included in the health packages under health insurance.

**Table 2.** Responses based on study year.

Characteristic	Study year			Total Median (IQR)	p value
	3 ^rd^	4 ^th^	Intern		
Oral health is an integral part of general health	5.0(4.0-5.0)	5.0(4.0-5.0)	5.0(4.0-5.0)	5.0(4.0-5.0)	0.382
Periodontitis is the 6 ^th^ complication of diabetes	4.0(3.0-4.0)	3.0(3.0-4.0)	3.0(3.0-4.0)	3.33(3.0-4.0)	0.341
oral check-up for all woman in pre-natal care	4.0(3.0-4.0)	4.0(3.0-4.0)	3.0(3.0-4.0)	3.66(3.0-4.0)	0.140
HIV pt. with CD4 count > 200 cells/mm blood is suitable for dental treatment	3.0(3.0-4.0)	3.0(3.0-4.0)	3.0(3.04-4.0)	3.0(3.0-4.0)	0.652
Salivary biomarkers used in diagnosis of oral and systemic diseases	4.0(4.0-5.0)	4.0(3.0-4.0)	3.0(3.0-4.0)	3.66(3.0-5.0)	0.003 *
Physician should advice and motivate their patients to undergo dental check-up regularly	5.0(4.0-5.0)	4.0(4.0-5.0)	4.0(4.0-5.0)	4.33(4.0-5.0)	0.013 *
Medical students should attend compulsory rotation in dentistry	4.0(3.0-4.0)	3.0(2.0-4.0)	3.0(3.0-4.0)	3.33(2.0-4.0)	0.017 *
Complete dental check-up should be covered in health insurance	4.0(4.0-5.0)	4.0(4.0-5.0)	5.0(4.0-5.0)	4.33(4.0-5.0)	0.665
Referral for dental check-up before any elective surgeries	4.0(3.0-4.0)	4.0(3.0-4.0)	4.0(3.0-4.0)	4.0(3.0-4.0)	0.656
Integral collaboration between medicine and dentistry	4.0(3.0-4.0)	4.0(3.0-4.0)	4.0(3.0-4.0)	4.0(3.0-4.0)	0.056
Interact with dental students and mutually exchange knowledge	4.0(3.0-4.0)	4.0(3.0-4.0)	4.0(3.0-4.0)	4.0(3.0-4.0)	0.084

* p=0.05.

Most students were aware about the necessity for interprofessional practice but only 61.8% agreed to the foster integral collaboration; 12.8% disagreed and the rest were unsure. Medical students (61%) gave a median score of 4, and agree that regular interaction is required with dental students to mutually exchange knowledge. Interestingly, 25% of them have replied neutrally, which again indicates a lack of interest with regards to the same.

A statistically significant difference based on age was seen ( Table 3) with p value of 0.014 when asked about relation of HIV patient and dental treatment. Participants belonging to 21-25 years age groups showed lesser knowledge regarding the same.

**Table 3.** Responses based on age group.

Characteristic	Age group			Total Median (IQR)	p value
	20 and below 20	21-24	Above 25		
Oral health is an integral part of general health	5.0	5.0(4.0-5.0)	5.0	5.0(4.0-5.0)	0.075
Periodontitis is the 6 ^th^complication of diabetes	4.0(3.0-4.0)	3.0(3.0-4.0)	3.0(3.0-4.0)	3.33(3.0-4.0)	0.056
oral check-up for all woman in pre-natal care	4.0(3.0-4.0)	4.0(3.0-4.0)	3.0(3.0-4.0)	3.66(3.0-4.0)	0.232
HIV pt. with CD4 count > 200 cells/mm blood is suitable for dental treatment	4.0(3.0-4.0)	3.0(3.0-4.0)	4.0(4.0-5.0)	3.66(3.0-5.0)	0.014 *
Salivary biomarkers used in diagnosis of oral and systemic diseases	4.0	4.0(3.0-4.0)	4.0(3.0-4.0)	4.0(3.0-4.0)	0.360
Physician should advice and motivate their patients to undergo dental check-up regularly	5.0(4.0-5.0)	4.0(4.0-5.0)	4.0(3.0-5.0)	4.33(3.0-5.0)	0.374
Medical students should attend compulsory rotation in dentistry	4.0(3.0-4.0)	3.0(2.0-4.0)	3.0(3.0-5.0)	3.33(2.0-5.0)	0.085
Complete dental check-up should be covered in health insurance	4.0(4.0-5.0)	4.0(4.0-5.0)	5.0(4.0-5.0)	4.33(4.0-5.0)	0.806
Referral for dental check-up before any elective surgeries	4.0(3.0-4.0)	4.0(3.0-4.0)	3.0(3.0-4.0)	3.66(3.0-4.0)	0.676
Integral collaboration between medicine and dentistry	4.0(3.0-4.0)	4.0(3.0-4.0)	4.0(3.0-4.0)	4.0(3.0-4.0)	0.191
Interact with dental students and mutually exchange knowledge	4.0(3.0-4.0)	4.0(3.0-4.0)	4.0(3.0-4.0)	4.0(3.0-4.0)	1.000

* p=0.05.

## Discussion

This study was used to evaluate the knowledge and attitudes of medical students towards the collaboration of medical and dental practice. As per Zhang the dental students would be more aware collaborative efforts than medical field as dental students are exposed to medical colleges from 1 ^st^ year to 3 ^rd^ year of their curriculum, contrary to the medical students who spend only a month in dental college postings.

Overall, medical students showed fairly good knowledge and positive attitude towards medical and dental collaboration in congruence with the results obtained from the study by Zhang. But analysis of groups within each parameter showed a significant difference. Based on year of study, it was found that students from third and final years of study had more positive attitudes than the interns, unlike results obtained by Zhang. ^7^ More than half the participants, particularly the interns, did not agree to attend compulsory rotation in dentistry (p value 0.017), contrary to finding in which Hendricson and Cohen concluded this rotation-ship was not only beneficial but essential. ^8^

Although nearly 50% participants had fair knowledge regarding the oral-systemic link, many participants were confused when asked if it was mandatory to undergo an oral check-up before pregnancy. Sufficient research has shown that severe periodontal disease in pregnant women predisposes them to a higher risk of delivering preterm and/or low-birth weight of the new born. ^9^^,^^10^ Offenbacher found mothers with periodontal disease are at a risk seven times more than mothers without. ^11^ When asked about a link between diabetes and oral health, students seemed to have limited knowledge regardless of year of study. In addition, previous investigations have established an association between either type 1 or type 2 diabetes and periodontal diseases to the extent that periodontitis has been called the “sixth complication of diabetes”. ^12^^,^^13^ Interestingly, analysis among gender revealed a statistically significant difference with more knowledge among female participants with regard to questions about criteria to undergo treatment among HIV patients. Though it does not provide any supporting evidence to prove poor knowledge, it does indicate the need for further education among medical students about HIV patients and dental treatment.

While assessing the attitude of the students, we found significant data that junior students advised and motivated their patients to undergo dental check-up regularly, compared to senior students who gave a more of neutral response. One of the reasons for such an attitude from senior students can be because of the concept of social hierarchy which can be due to lack of interprofessional communication and patient management. ^4^

In the United States, utilization of oral health care services and the incidence of oral disease are strongly linked to dental insurance coverage. ^14^ In contrast, in India the dental insurance sector is less prevalent, 40-50% of the medical students strongly feel that dental check-up and some part of treatment must be covered in general health packages.

Around 60% of participants responded positively towards the integral collaboration and interprofessional communication, although 30% students were not sure and the rest disagreed with it. Analysis showed that the third and final year students were more positive than interns which is in contrast to results obtained from a study by Zhang. ^7^ The exposure medical students undergo at clinics along with their interest in the subject affects their perception of oral health and its importance on general health. The Indian health education system, which often displays egocentric power relations among healthcare professionals, whereby medical professionals may not consider oral health as an integral part of general health due to a false perception, is threatening this interprofessional collaboration. ^15^

Students’ attitude is associated with factors such as gender, knowledge of regular dental check-up, and curriculum. Results of a previous study reported that gender could affect a student’s attitude towards medical dental collaboration. ^9^^,^^16^ Questions pertaining to the attitude towards collaboration such as insurance benefits for dental treatments received a more positive response from females than males. When asked about importance of interprofessional communication for exchange of knowledge and better patient care, females gave a greater positive response than males, which can be attributed to higher ego among males. ^15^

In clinical practice, interprofessional continuing education is a useful means of regulating and stabilizing a professional’s identity and improving teamwork. ^4^ Guidelines must be set to improve confidence in a provider’s ability with regard to cases pertaining to both fields and have access to updated knowledge about the collaboration between medical and dental practice. ^17^^,^^18^ The existing body of medical and dental professionals play an important role since they have the ability to lay the guidelines. They can set guidelines for the indications, timing, protocols, and responsibilities of referral and consultation among physicians and dentists. Patients and the community should be made to understand the relationship between oral and systemic health by means of awareness campaigns. ^19^ In doing so, national health goals can be achieved by reducing these kinds of healthcare disparities.

Apart from this, studies with larger sample size should be considered in future, to extrapolate the study results to a larger professional population. Even para-medical healthcare providers can be included as a part of the study to seek better understanding.

## Limitations of the study

While we attempted to assess the attitude and knowledge of medical students, there are few confounding factors which may affect the responses of the participants such as participants personal interest in the subject, Interaction with para medical and dental peers which may affect their perception. Future studies can be structured to eliminate such bias.

## Conclusion

• Medical students showed fairly good knowledge and positive attitude towards dentistry, the analysis within the study groups showed that knowledge and attitude regarding the collaborative practice declined over the academic years among the medical students.
• There is a need for improved healthcare education curriculum for interprofessional management of patients with stress on significance of effects of oral health.

## Data availability

### Underlying data

figshare: HARSHIT Data MASTERCHART.xlsx. https://doi.org/10.6084/m9.figshare.19409354.v2 ^20^

This project contains the following underlying data:

- Data chart.xlsx

### Extended data

figshare: HARSHIT Data MASTERCHART.xlsx. https://doi.org/10.6084/m9.figshare.19409354.v2 ^20^

This project contains the following extended data:

- Data code statistical analysis.xlsx
- Questionnaire with consent form.docx

Data are available under the terms of the Creative Commons Zero “No rights reserved” data waiver (CC0 1.0 Public domain dedication).
