# Supplementary material for: Attitudes toward and knowledge of collaboration of dental and medical practice among medical students in Southern India: a cross-sectional questionnaire survey
Source: F1000Res. 2023 Jun 27;11:476. Originally published 2022 Apr 29. [Version 4] doi: 10.12688/f1000research.111130.4 (PMC10794861; doi:10.12688/f1000research.111130.4)
Supplement: Peer review history

# Reviewer report: Reviewer response for version 4

**DOI:** 10.5256/f1000research.151858.r234387

**Published:** 2024-01-17

**Authors:** Mahesh Khairnar, Umesh Wadgave

**Affiliations:** 1 Banaras Hindu University, Varanasi, India; 2 Bharati Vidyapeeth Dental College, Pune, Maharashtra, India

1. How was the sample size calculated?
2. What were the results of content validation?
3. Was the reliability of the questionnaire tested?
4. Check the sentences for language errors. Past tense should be used while framing the sentence in the manuscript.
5. What was the purpose of categorizing age into three different categories? Does it make any sense? The age groups were closely related (<20,20-25,>25).
6. The authors have not presented the reasoning for questions showing significant differences.

Is the work clearly and accurately presented and does it cite the current literature?

Yes

If applicable, is the statistical analysis and its interpretation appropriate?

Partly

Are all the source data underlying the results available to ensure full reproducibility?

Yes

Is the study design appropriate and is the work technically sound?

Yes

Are the conclusions drawn adequately supported by the results?

Yes

Are sufficient details of methods and analysis provided to allow replication by others?

Yes

Reviewer Expertise:

Public Health Dentistry

We confirm that we have read this submission and believe that we have an appropriate level of expertise to confirm that it is of an acceptable scientific standard, however we have significant reservations, as outlined above.

# Reviewer report: Reviewer response for version 4

**DOI:** 10.5256/f1000research.151858.r181922

**Published:** 2023-07-04

**Authors:** Pathiyil Ravi Shankar

**Affiliations:** 1 IMU Centre for Education, International Medical University, Kuala Lumpur, Malaysia

I have no further comments

Is the work clearly and accurately presented and does it cite the current literature?

Partly

If applicable, is the statistical analysis and its interpretation appropriate?

Yes

Are all the source data underlying the results available to ensure full reproducibility?

Yes

Is the study design appropriate and is the work technically sound?

Partly

Are the conclusions drawn adequately supported by the results?

No

Are sufficient details of methods and analysis provided to allow replication by others?

Partly

Reviewer Expertise:

Health professions education

I confirm that I have read this submission and believe that I have an appropriate level of expertise to confirm that it is of an acceptable scientific standard.

# Reviewer report: Reviewer response for version 3

**DOI:** 10.5256/f1000research.151361.r178018

**Published:** 2023-06-13

**Authors:** Pathiyil Ravi Shankar

**Affiliations:** 1 IMU Centre for Education, International Medical University, Kuala Lumpur, Malaysia

The authors have tried to address some of the points I raised during the previous review. 

 The points still unclear to me are the pretesting of the questionnaire and the percentage of students from each college who participated in the study. How exactly was the questionnaire administered? Did students feel obliged to participate in the study? I am unable to understand the point about continuing dental education mentioned in the Conclusion section. Also only having postings of dental students in medical departments may not lead to lead to a positive attitude toward collaboration unless it is incorporated into the curriculum.

Is the work clearly and accurately presented and does it cite the current literature?

Partly

If applicable, is the statistical analysis and its interpretation appropriate?

Yes

Are all the source data underlying the results available to ensure full reproducibility?

Yes

Is the study design appropriate and is the work technically sound?

Partly

Are the conclusions drawn adequately supported by the results?

No

Are sufficient details of methods and analysis provided to allow replication by others?

Partly

Reviewer Expertise:

Health professions education

I confirm that I have read this submission and believe that I have an appropriate level of expertise to confirm that it is of an acceptable scientific standard, however I have significant reservations, as outlined above.

# Reviewer report: Reviewer response for version 2

**DOI:** 10.5256/f1000research.137907.r149770

**Published:** 2022-09-27

**Authors:** Pathiyil Ravi Shankar

**Affiliations:** 1 IMU Centre for Education, International Medical University, Kuala Lumpur, Malaysia

Thank you for the invitation to review this manuscript.

 My suggestions are:

**Introduction**

- The authors can highlight what learning and other initiatives are undertaken at their institution to promote interprofessional education and collaboration.
- Why did the authors not study the attitude of dental students? For collaboration, both parties involved should have a favorable attitude.

**Methods**

- Ethical approval was obtained over five years ago in 2017. What is the duration of the approval provided by the ethical committee?
- The study was done in 2019. It may be interesting to study the impact of COVID-19 on student attitudes.
- How exactly was the data collected? Where were the questionnaires completed?
- I am confused about which colleges were involved in the study. What percentage of students from each college had participated? How were these colleges selected?
- Page 8: Can the authors clarify the statement that they avoided including dental students to avoid positive response bias?
- In the limitations section, can the authors elaborate on how upbringing can affect interprofessional collaboration?
- Many of the statements in the questionnaire deal with dental complications of diseases and are not related to collaboration between medical and dental practitioners in my opinion. Can more details about the validation process of the questionnaire be provided?

**Conclusion**

- The conclusion about continuing dental education does not seem to be based on data gathered during the study.
- A big limitation is not including the dental students and not having any idea about their perception of collaborative care.

Is the work clearly and accurately presented and does it cite the current literature?

Partly

If applicable, is the statistical analysis and its interpretation appropriate?

Yes

Are all the source data underlying the results available to ensure full reproducibility?

Yes

Is the study design appropriate and is the work technically sound?

Partly

Are the conclusions drawn adequately supported by the results?

No

Are sufficient details of methods and analysis provided to allow replication by others?

Partly

Reviewer Expertise:

Health professions education

I confirm that I have read this submission and believe that I have an appropriate level of expertise to state that I do not consider it to be of an acceptable scientific standard, for reasons outlined above.

# Response

**Published:** 2022-10-20

**Authors:** Harshit Atul Kumar

**Affiliations:** Manipal College of Dental Sciences,Mangalore, India

Thank you for the suggestions.

 I would like to share with the reviewer that in India dental schools are not involved in teaching oral or dental healthcare-related topics among medical students. As per the curriculum, medical students have approximately a month of internship in dental school to shadow a dental faculty to observe cases. On the contrary, dental students are actively taught by faculty from the medical college and even have clinical postings for an entire year. Hence, the reason for not including dental students in this survey was that they have more exposure to possible interdisciplinary collaboration when compared to medical students. Although this doesn't guarantee a positive bias, the questions designed for the questionnaire are pertaining to how oral health affects general health, for which, we decided to assess medical students for their knowledge, attitude, and practice and avoided the dental students.

 Ethical approval was obtained in 2017 and the study was completed in 2019, once the study was completed, we submitted the report to the institutional ethical committee, after which the validity of ethical approval is over. 

 I have mentioned in the methodology section about how and where the data was collected. We visited the medical colleges in and around Mangalore and collected responses in pen and paper format to meet the calculated sample size.

# Reviewer report: Reviewer response for version 1

**DOI:** 10.5256/f1000research.122818.r136383

**Published:** 2022-07-26

**Authors:** Saba Kassim

**Affiliations:** 1 Department of Preventive Dental Sciences,College of Dentistry, Taibah University, Medina, Saudi Arabia

Thank you for considering me to review this paper.

 My comments are as follows:

1. If the questionnaire was validated elsewhere, why was content and face validity done? By convention reliability (Cronbach alpha) and construct validity should be tested.
2. The authors repeated that the students were assessed for their knowledge and attitude using a 5-point Likert scale. Please check the last two paragraphs under ‘Study and questionnaire design’.
3. The authors calculated the mean response score for each question and conducted the statistical analysis with the median of scores received as a measure of central tendency and evaluated the statistical significance. Why were the mean and median calculated at the same time? One of these central tendencies should be used according to the normality distribution. In addition, the authors compare the three age groups and education levels and mentioned the wrong test, Mann Whitney U test. It was appropriate to use ANOVA (if scores are normally distributed) with post hoc analysis to compare the three groups.
4. The authors used 'interdisciplinary' and 'interprofessional' interchangeably, consistency in using one of these terms is preferable unless there was an obvious reason(s).
5. This study has a number of limitations that should be mentioned in the discussion section.

Is the work clearly and accurately presented and does it cite the current literature?

Yes

If applicable, is the statistical analysis and its interpretation appropriate?

Partly

Are all the source data underlying the results available to ensure full reproducibility?

Yes

Is the study design appropriate and is the work technically sound?

Partly

Are the conclusions drawn adequately supported by the results?

Yes

Are sufficient details of methods and analysis provided to allow replication by others?

Partly

Reviewer Expertise:

Dentistry

I confirm that I have read this submission and believe that I have an appropriate level of expertise to confirm that it is of an acceptable scientific standard, however I have significant reservations, as outlined above.

# Response

**Published:** 2022-08-03

**Authors:** Harshit Atul Kumar

**Affiliations:** Manipal College of Dental Sciences,Mangalore, India

Thank you very much for your valuable review. I appreciate your effort to review my article. I understand the points you have raised and I shall make amendments and bring more clarity to the article. Article shall be updated accordingly and resubmitted soon.

# Response

**Published:** 2023-05-05

**Authors:** Harshit Atul Kumar

**Affiliations:** Manipal College of Dental Sciences,Mangalore, India

I have updated the article as per reviewers suggestions to Version 1 & 2 . I kindly request the reviewers to re-review again.
